# An Optimum Deployment Algorithm of Camera Networks for Open-Pit Mine Slope Monitoring

**DOI:** 10.3390/s21041148

**Published:** 2021-02-06

**Authors:** Hua Zhang, Pengjie Tao, Xiaoliang Meng, Mengbiao Liu, Xinxia Liu

**Affiliations:** 1 School of Remote Sensing and Information Engineering, Wuhan University, Wuhan 430079, China; hzhang_rs@whu.edu.cn (H.Z.); pjtao@whu.edu.cn (P.T.); mengbiaoliu@whu.edu.cn (M.L.); 2 School of Water Conservancy and Electric Power, Hebei University of Engineering, 62#Zhonghua Street, Handan 056038, China; liuxinxia@hebeu.edu.cn

**Keywords:** camera networks, open-pit mine slope monitoring, optimum deployment, close range photogrammetry, three-dimensional reconstruction, OCD4M

## Abstract

With the growth in demand for mineral resources and the increase in open-pit mine safety and production accidents, the intelligent monitoring of open-pit mine safety and production is becoming more and more important. In this paper, we elaborate on the idea of combining the technologies of photogrammetry and camera sensor networks to make full use of open-pit mine video camera resources. We propose the Optimum Camera Deployment algorithm for open-pit mine slope monitoring (OCD4M) to meet the requirements of a high overlap of photogrammetry and full coverage of monitoring. The OCD4M algorithm is validated and analyzed with the simulated conditions of quantity, view angle, and focal length of cameras, at different monitoring distances. To demonstrate the availability and effectiveness of the algorithm, we conducted field tests and developed the mine safety monitoring prototype system which can alert people to slope collapse risks. The simulation’s experimental results show that the algorithm can effectively calculate the optimum quantity of cameras and corresponding coordinates with an accuracy of 30 cm at 500 m (for a given camera). Additionally, the field tests show that the algorithm can effectively guide the deployment of mine cameras and carry out 3D inspection tasks.

## 1. Introduction

Slope damage results in serious disasters that cause thousands of deaths and injuries and extensive property damage every year [1]. This poses a serious threat to people working in open-pit mines with slopes. There are three scales of slope damage that can occur in open-pit slopes, and they are bench damage, interslope damage and overall damage [2]. With economic development and the rapid growth of the demand for mineral resources, the exploitation of mine enterprises continues to increase. Many hillside open-pit mines are transformed into deep mines, which leads to increasing the overall angles of slopes and consequently, an increased landslide risk [3]. The statistics of industrial accidents that occurred during the 2005–2010 open-pit coal production period in Turkish coal companies indicates that the most likely risks in open-pit mines are related to mine slopes [4]. According to the US Centers for Disease Control and Prevention (CDC) statistics for mine disasters in 2014, mine slope-related accidents were most reported in quarry operations, accounting for 33.3% of all accidents [5]. In total, 40% of Chinese open-pit mines have slope stability problems. According to statistics, there were 240 slope collapses and 369 fatalities from 2013 to 2017, ranking second amongst noncoal mine accidents in China [6]. Consequently, it is especially important to monitor the slope of open-pit mines.

For mine slope safety monitoring, scholars have used geodetic methods, 3S technology, photogrammetry, the Synthetic Aperture Radar (SAR), 3D laser scanning, and other methods in associated research. The geodesic survey methods [7], such as leveling instrument, theodolite, rangefinder, and distance measurement equipment, represent technology which is widely used in the establishment of high-precision control networks of mines [8]. In addition, the high-precision deformation monitor is mature, displays data reliability, and has a high accuracy; however, for open-pit mine slope monitoring, it has the disadvantages of requiring a large degree of manual involvement, being influenced by terrain access and climate, and is not able to automate monitoring, and those disadvantages result in low detection efficiency. Scholars have begun to focus more on other techniques to carry out mine slope safety monitoring. Manconi et al. [9] used surveying robot data to monitor the mine slope and simplify the complex deformation of the slope, which gained support from relevant departments due to good experimental results. Wang et al. [10] integrated Global Positioning System (GPS)/pseudo-satellite (PL) positioning technology to improve the position settlement accuracy and provide a high-precision monitoring model for high-precision slope monitoring in open-pit mines. Akbar et al. [11] employed the integration of GPS, the Geographic Information System (GIS), and Remote Sensing (RS) to map a hill slide disaster map. Zeybek et al. [12] used a long-range terrestrial laser scanner to measure the precision of Taşkent Landslide (Konya, Turkey). Liao et al. [13] applied high-resolution SAR data to monitor landslides in the Three Gorges Reservoir area in China and were able to identify the precise location, deformation, and time range of the landslide more accurately. Tang et al. [14] used new generation SAR satellites (Sentinel-1 and TerraSAR-X) to map surface displacements and slope instability at three open-pit mines in the Rhenish coalfield in Germany, in order to provide a long-term monitoring solution for open pit mining and its operations. Wang et al. [15] employed inclined photogrammetry to generate a mine Digital Surface Model (DSM) and carried out the construction of a Digital Elevation Model (DEM) for an open-pit mine. Alameda-Hernández et al. [16] used ultraclose range terrestrial digital photogrammetry to monitor the stability of soft foliated rocky slopes and analyzed their errors during rock weathering using the example of soft rocks in Alpujarras (Andalusia, Spain). Tong et al. [17] used Unmanned Aerial Vehicle (UAV) photogrammetry and ground-based laser scanning for open-pit mine inspection and three-dimensional (3D) mapping, aligning image data with point cloud data and classifying land cover. González-Díez et al. [18] elaborated on the methods which used digital photogrammetry to accurately measure slope changes caused by landslides.

In consideration of the use of each monitoring method and the research results of the above-mentioned references, the characteristics of each monitoring method and its corresponding scope of application are summarized in Table 1. Among the methods applied for open-pit mine monitoring, the close-up photogrammetry technique is a moderate method which can provide high efficiency and inexpensive measurement, especially compared to the other usual methods of laser scanning, the Interferometric Synthetic Aperture Radar (InSAR), the Laser Radar (LiDAR), etc. [19,20,21]. The use of photogrammetry has the requirement of capturing images with a degree of overlap, which places demands on the deployment of cameras.

The visual camera network, as a type of sensor network, is a spatially distributed network of smart cameras that collects and processes multimedia information to transform scene images into a more useful form [22]. Visual sensors can perceive more information than ordinary sensors, and visual sensor networks can handle higher-level visual tasks than single vision sensors [23]; for example, Kulkarni et al. [24] designed the multilayer camera network sensEye for object monitoring, identification, and tracking. In addition, coverage is an important aspect when evaluating the quality of detection of multiple regions of interest in visual sensor networks and is an important research direction for camera networks. Related studies on the coverage problem of visual sensor networks have been conducted and relevant algorithms have been designed to obtain the maximum coverage units with the most optimal camera deployment scheme [25,26]. Based on the research of visual sensor networks, it is a good choice to introduce the idea of visual sensors in the field of slope risk monitoring; make full use of the multimedia resources of the camera; and realize functions such as 3D slope monitoring, tramcar positioning, and video monitoring.

The intelligent application of multicamera video data in mining is an important aspect of smart mine constructions, and a number of studies have applied photogrammetry to the digitization of mines [27], reconstructing visual data in three dimensions and measuring parameters such as slope deformation monitoring and the slope gradient. Giacomini et al. [21] used close-up photogrammetry to continuously monitor the rock surface, in order to assess the potential rockfall risk and estimate the area of impact. Aggarwal et al. [28] implemented an Internet of Things (IOT) landslide monitoring system based on Raspberry Pi using a camera. It analyzed the area in real time based on the video stream obtained from the camera and applied computer vision algorithms to detect landslides. This method can only monitor the occurrence of large landslides and cannot provide an early warning, and the distance cannot be too far. A camera can also be mounted on a UAV to reconstruct the mine pit in 3D to obtain a DSM by extracting the comparative elevation, slope, slope direction, surface fluctuations, and surface roughness distribution and performing crack analysis [29,30,31]. Kromer et al. [32] used a digital Single Lens Reflex (SLR) camera to form a camera system. The use of photogrammetric workflows for mine slope monitoring achieved a high level of accuracy, but the shortcoming of this paper is that it does not describe the camera network deployment options for mine conditions and camera parameters, as well as the corresponding budgets. The intelligent use of video camera data at a mine site for slope collapse risk monitoring is a trend in smart mine constructions, and the rationalization of camera deployment is one of the most important aspects. At the present stage, corresponding optimized camera network deployment rarely occurs in open-pit mines. 

To solve the above problem, we propose an optimum deployment algorithm of camera networks for open-pit mine slope monitoring. The remainder of the paper is structured as follows: in Section 2, the optimum deployment algorithm for open-pit mine landslide monitoring is presented; in Section 3, the experimental simulation for the algorithm, field test, and demonstration are described; and finally, in Section 4, the discussions and conclusions are provided.

## 2. Materials and Methods

**Figure 1:**
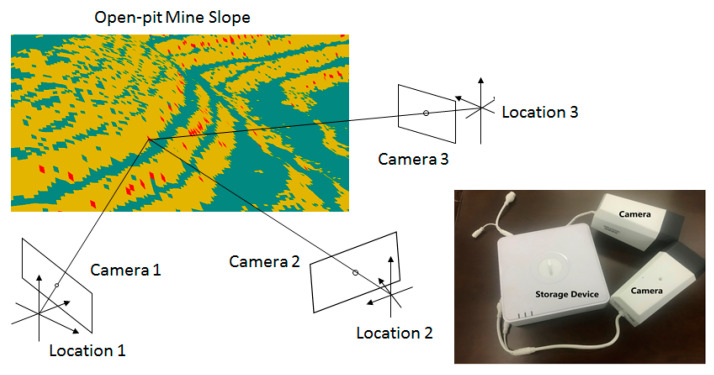
Sketch deployment diagram for open-pit mine slope monitoring. At the top left, the blue area represents the bench work surface, the yellow area represents the bench slope and the red area represents the risk exceeding the threshold.

This study introduces the idea of combining visual camera observation with digital photogrammetry, and designs the Optimum Camera Deployment algorithm for open-pit mine slope monitoring (OCD4M). For a given mine slope, an observation platform, and camera parameters, the algorithm determines the deployment scheme with the minimum quantity of cameras and the optimal camera positions, in order to meet the need for overlap in 3D monitoring by close-up photogrammetry and the need for full coverage of safety monitoring, as shown in a simple deployment schematic in Figure 1. In this section, we introduce the OCD4M algorithm in terms of monitoring object description, mathematical model, mine surface preprocessing, and deployment algorithm workflow.

### 2.1. Description of Targets and Criteria for Monitoring

**Figure 2:**
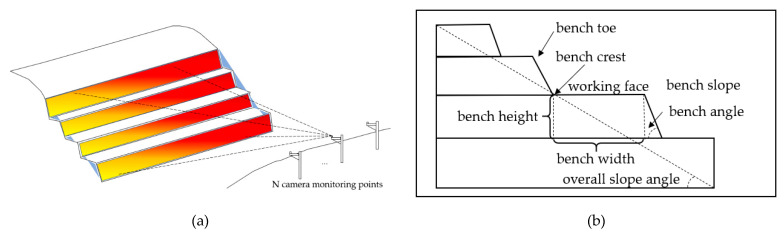
Schematic diagram of the monitoring object. (**a**) Schematic diagram of the monitoring of the open pit slope by cameras. (**b**) Section of the mine slope with the various parts of the open-pit mining.

Figure 2 shows the composition of the mine’s slope. The object of monitoring is to calculate the bench width, bench height, bench slope angle and overall slope angle of the open-pit slope, in order to meet the safety requirements, and to provide early warning if the threshold values are exceeded. The calculation accuracy is required to be decimeter.

### 2.2. Model Description and Problem Definition

**Figure 3:**
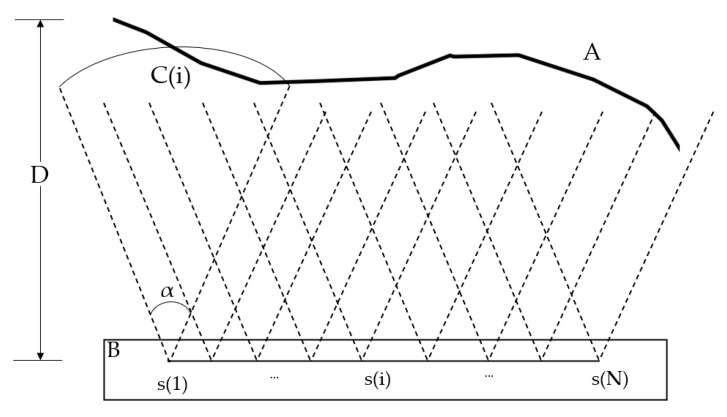
Geometric deployment of the camera sensor network, where curve A is the surface, B is the observation platform, s(i) is the camera sensor, the area corresponding to the two dashed lines is the shooting range C(i), α is the field of view, and D is the shooting distance.

As shown in Figure 3, the camera sensor network contains a number (N) of sensors (S), which are deployed at the observation platform (B) that monitors the target surface (A).

(1) $$S=\{s_{1},\;s_{2},\;\ldots ,\;s_{N}\},\;A\;:\;f(x,\;y,\;z)=0,\;B\;:\;g(x,\;y,\;z)=0$$

From a geometric point of view, for each sensor, its sensing area is defined by tuple ${C(i):\;(s}_{i}\left(x,\;y,\;z),\;D,\;\alpha ,\;\theta \right)$, ${\mathrm{where}\;s}_{i}\left(x,\;y,\;z\right)$ is the sensor’s position, D is the photography distance, α is the sensing angle, and θ is the angle at which the camera deviates from the normal case photography direction. The aims are to ensure that the target surface is fully covered by the sensing area and that the image overlap rate between adjacent sensors is more than 80%. We can define the aims by the following three formulas:

(2) $$A\subseteq C_{s\left(1\right)}\cup C_{s\left(2\right)}\cup \ldots \cup C_{s(N)},$$

(3) $$S_{C_{s\left(i\right)}\cap C_{s\left(i+1\right)}}\geq 0.8S_{C_{s\left(i\right)}},$$

(4) $$s_{i}\left(x,\;y,\;z\right)\subseteq B,$$

### 2.3. Discussion of Different Situations

**Figure 4:**
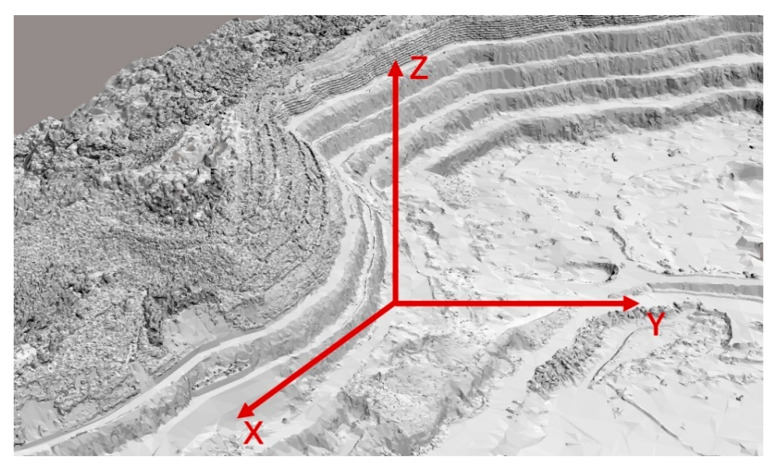
Coordinate system established within the mine.

The mine coordinate system is established as shown in Figure 4, with the vertical direction as the *Z*-axis, the photographic direction as the *Y*-axis, and the plane perpendicular to the plane formed by Z and Y as the *X*-axis. 

**Figure 5:**
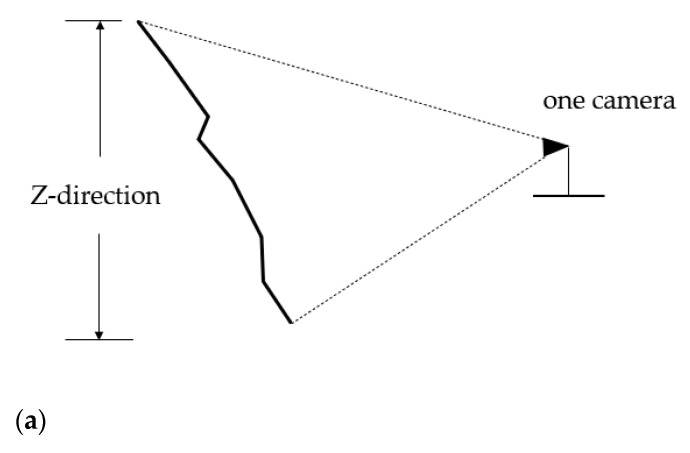

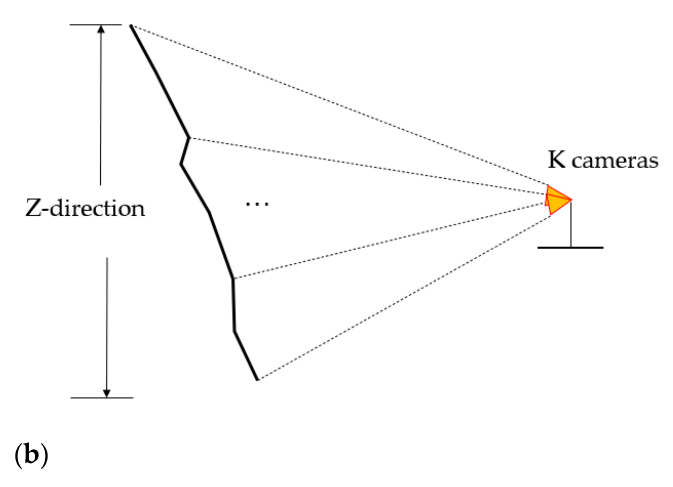
Observation coverage in the Z-direction. (**a**) The case where a single camera can cover the Z-direction of the slope of the open-pit mine, where a single camera is deployed on the right, with the dashed line showing the range of shots and the black curve showing the Z-direction of the mine slope. (**b**) The case where K cameras cover the Z-direction of the broken face of the mine, similar to (**a**), where multiple cameras are deployed on the right, with multiple dashed lines and ellipses indicating the range of the multiple cameras.

In order to sense the whole Z-direction (Figure 4) area, one or more cameras need to be deployed. As shown in Figure 5a, when the sensing area of a single camera can cover the Z-direction of the mine face, only one camera is sufficient at this point; if not, multiple K cameras as shown in Figure 5b need to be deployed at the same monitoring point to meet the need for sensing the whole coverage of the Z-direction, while the overlap of the monitoring areas of the multiple K deployed cameras also needs to be satisfied to meet the photogrammetry requirements.

**Figure 6:**
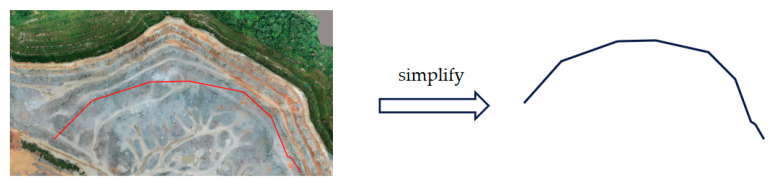
Mine surface simplification: in the XY flat, closest to the camera is the slope line where the red curve in the diagram is located. The closer the distance, the smaller the camera’s sensing range. If this line can be fully covered, other heights of the slope must be covered, so we have chosen this line to represent the situation of the mine surface in the XY plane.

In the XY flat area, we simplify the mine surface. In consideration of the complexity of the stereoscopic surface, for the purpose of computational convenience, the mining surface is simplified twice. The slope toe of the mine is closer to the observation platform. In terms of the characteristics of the camera sensors, the closer the observation is, the smaller the observation range. Consequently, we can simplify the surface at Figure 6 to form a curve which is located at the bottom of the mine.

**Figure 7:**
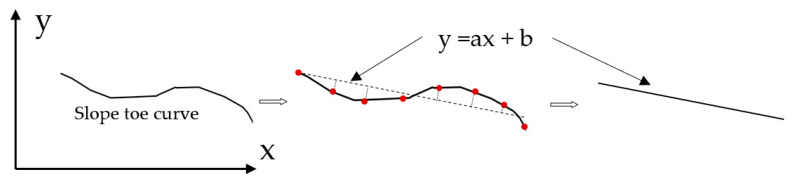
Slope toe simplification: the diagram shows how the broken line is finally reduced to a straight line by line fitting.

In order to reduce the complexity of the calculation, we use a straight line instead of the curve obtained by simplifying Figure 7. The specific approach was carried out using least squares to find the best-fitting straight line. This is modeled by selecting several points on the curve at certain intervals, which are used to solve the linear equation:

(5) y=ax+b,

for the parameters a and b, where a and b are calculated by the following equations:

(6) $$a=\frac{\mathrm{Num}\sum x_{i}y_{i}-\sum x_{i}\sum y_{i}}{\mathrm{Num}\sum {x_{i}}^{2}-(\sum x_{i}{)}^{2}},\;b=\frac{\sum y_{i}\sum {x_{i}}^{2}-\sum x_{i}\sum x_{i}y_{i}}{\mathrm{Num}\sum {x_{i}}^{2}-(\sum x_{i}{)}^{2}},\;i=1,\;2,\;\ldots ,\;\mathrm{Num},$$

where Num is the number of data points (red points in Figure 7).

**Figure 8:**
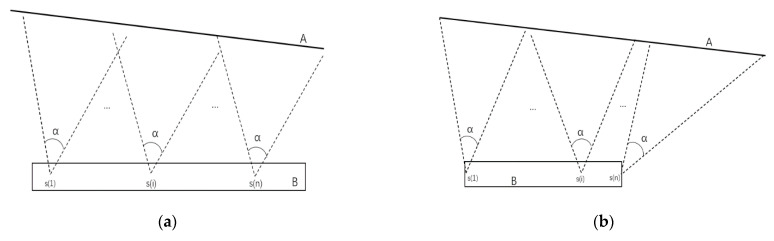
Camera sensor deployment in the X-direction: (**a**) the use of normal case photography and (**b**) the use of convergent photography to cover the entire mine surface. A represents the observed mine surface, the dotted line represents the photographic area, α represents the field of view, s(i) represents the camera monitoring point, and B represents the observation platform.

According to the observation platform and mine surface, it can be divided into two situations, as Figure 8 shows. In the X-direction, when the length of the observation is longer than the mine surface, we adopt normal case photography; otherwise, we adopt convergent photography, which has a larger coverage area, in order to meet the requirement.

### 2.4. The Optimum Deployment Algorithm

**Figure 9:**
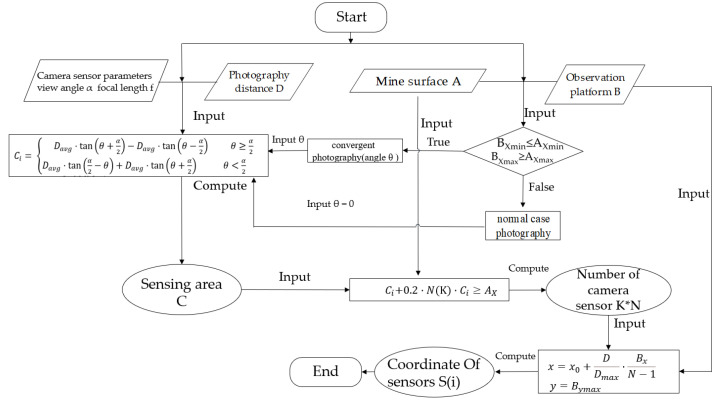
Workflow of the Optimum Camera Deployment algorithm for open-pit mine slope monitoring (OCD4M) algorithm.

The OCD4M algorithm is used to solve the camera sensor deployment problem. The algorithm starts with the input of camera sensor parameters, mine face parameters, observation platform parameters, and photographic distances. The next step is to simplify the corresponding mine face in conjunction with the mining extent. Next, we calculate the minimum quantity of cameras and the coordinates through the algorithm. Therefore, the workflow of the algorithm is as shown in Figure 9.

The input parameters for the algorithm are the parameters of the camera sensors (mainly the camera’s field of view α and focal length f), the shooting distance D, the mine plane A to be observed, and the observation platform B. The sensing range C of the camera sensor can be calculated from the camera parameters and the shooting distance using the following formula:

(7) $$C_{i}{=2\cdot D}_{\mathrm{avg}}\cdot \tan\frac{\alpha }{2}.$$

In the above equation, C_i_ is the camera area, including C_ix_ and C_iz_; D_avg_ is the camera distance; and α is the field of view of the camera.

The next step calculates the number of cameras K per surveillance point to achieve full coverage of the open-pit slope in the Z-direction, calculated according to the following formula:

(8) $$C_{iz}+0.2\cdot K\cdot C_{iz}\geq A_{Z}$$

where C_iz_ is the length of the photographic area C_i_ in the Z-direction, K is the quantity of cameras, and A_z_ is the extent of the mine slope in the Z-direction.

When the X-direction of the open-pit mine slope A is greater than the X-direction range of the observation platform B, normal case photography is used, and the minimum quantity of monitoring points N is calculated according to the following equation:

(9) $$C_{ix}+0.2\cdot N\cdot C_{ix}\geq A_{X}.$$

In the above equation, C_i__x_ is the camera area in the X-direction, N is the minimum quantity of monitoring points, and A_x_ is the extent of the mine slope in the X-direction.

Otherwise, convergent photography is used, and the minimum quantity of cameras N can be obtained from

(10) $$C_{i}=\{\begin{array}{c} D_{\mathrm{avg}}\cdot \tan\left(\theta +\frac{\alpha }{2}\right){-D}_{\mathrm{avg}}\cdot \tan\left(\theta -\frac{\alpha }{2}\right)\;\theta \geq \frac{\alpha }{2}\\ D_{\mathrm{avg}}\cdot \tan\left(\frac{\alpha }{2}-\theta \right){+D}_{\mathrm{avg}}\cdot \tan\left(\theta +\frac{\alpha }{2}\right)\;\theta <\frac{\alpha }{2} \end{array}.$$

In the above equation, C_i_ is the camera area in the X-direction, D_avg_ is the camera distance, α is the field of view of the camera, θ is the angle of deviation with respect to the orthogonal direction, and A_x_ is the extent of the mine slope in the X-direction. The range of cameras $C_{\min}{\sim C}_{\max}$ is calculated to solve $N_{\min}{\sim N}_{\max}$ according to Formula (9). Based on the results of the quantity of cameras, the coordinates of each camera on the mining plane can be calculated through the following formula:

(11) $${x=x}_{0}+\frac{D}{D_{\max}}\cdot \frac{B_{x}}{N-1},{\;y=B}_{\mathrm{ymax}},$$

where y is the maximum value employed to achieve greater coverage, and the quantity of cameras is K*N.

The whole workflow of the Algorithm 1 can also be described by the following pseudocodes.
**Algorithm 1.** OCD4M**Require:**Camera sensor set ${S=\{s}_{1}{,s}_{2}{,\ldots ,s}_{N}\}$, target surface A:f(x,y,z)=0, Observation platform B:g(x,y,z)=0, Photography distance D, sensing angle α**Ensure:**A is covered by the set of $C(i):(\mathrm{xyz}(s(i)),\;D,\;\alpha ,\vec{d_{i}})$, and the degree of overlap of adjacent C(i) greater than 80%. (1) $A\subseteq C_{s\left(1\right)}\cup C_{s\left(2\right)}\cup \ldots \cup C_{s(N)}$; (2) $S_{C_{s\left(i\right)}\cap C_{s\left(i+1\right)}}\geq 0.8S_{C_{s\left(i\right)}}$; (3) ${\mathrm{xyz}(s}_{i})\subseteq B$.**Process:**1: Compute the range of A. $A_{Z}{=A}_{\mathrm{zmax}}{–A}_{\mathrm{zmin}}{;\;A}_{X}{=A}_{\mathrm{xmin}}{–A}_{\mathrm{xmax}}$,2: Compute the range of B. $B_{Z}{=B}_{\mathrm{zmax}}{–B}_{\mathrm{zmin}}{;\;B}_{X}{=B}_{\mathrm{xmin}}{–B}_{\mathrm{xmax}}$,3: Compute the rang of Ci,4: Whether to cover the Z direction of Mine Surface A5: while $C_{z}{*K<A}_{Z}$ do6:    get the number of camera sensors at each point7:    K++8: End while9: Judge the Length relationship of A and B10: If ($B_{\mathrm{Xmin}}\leq A_{\mathrm{Xmin}}{\;\mathrm{and}\;B}_{\mathrm{Xmax}}\geq A_{\mathrm{Xmax}}$) do11:    normal case photography, compute the minimum of camera sensors by Formula (9): 12:    Compute the coordinate of each camera sensor according to the photography13:    distance by Equation (11),14:    ${y=B}_{\mathrm{ymax}}$, z15: Else16:    convergent photography (angle θ);17:    compute the range of camera sensor number by18:    Equations (9)–(11),19:    we can get K, N and ${(s}_{i}\left(x,\;y,\;z)\right)$20: End If21: Return number (K*N) and position of sensor ${(s}_{i}\left(x,\;y,\;z)\right)$


## 3. Implementation and Results

### 3.1. Simulation of Experimental Tests

In this section, the results of the simulation experiments are given. The experimental tests focus on simulating the quantity of cameras and camera accuracy at different distances and the quantity of cameras and camera accuracy at different field of view angles. For small to medium-sized open-pit mines and some large mines, 500 m is relatively adequate. A greater distance means a lower accuracy, so beyond 500 m, it is necessary to improve the quality of the camera or add other means to control the accuracy, which means an increase in cost. This is unacceptable for small to medium-sized mines with low revenues, so we chose 500 m as the camera distance for our simulations. The main test condition parameters are as follows:

- Camera parameters
  Sensor size: 1/3” inches (4.8 mm × 3.6 mm)
  Focal length: 8 mm
- Ore surface parameters
  $A_{Z}$ = 100 m
  $A_{X}$ = 500 m
- Observation platform parameter
  $B_{X}$ = 500 m

#### 3.1.1. Quantity and Precision Analysis

**Figure 10:**
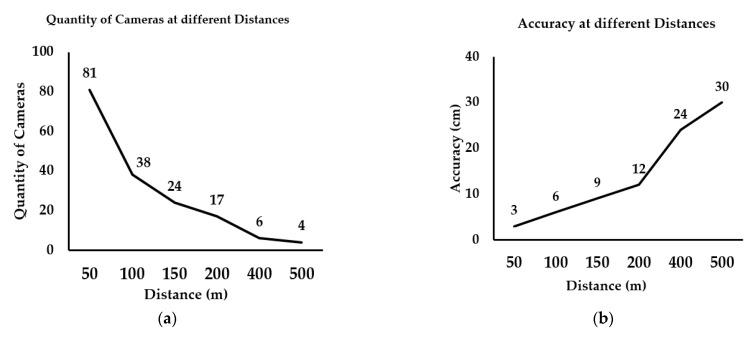
Quantity and accuracy of cameras at different distances. (**a**) The minimum quantity of cameras calculated for different distances. The further the distance, the fewer the cameras needed. (**b**) The distance each pixel represents in the field increases, which means that the accuracy decreases.

The camera focal length was 8 mm, the horizontal field of view angle was 32.69°, and the vertical field of view angle was 24.81°. Assuming that the monitoring distance was between 50 and 500 m, the minimum quantity of cameras and the resolutions were calculated as shown in Figure 10.

As shown in Figure 10a, according to Equation (7), the coverage becomes larger as the distance becomes larger whilst the camera remains the same, which means that the entire mine surface can be covered using fewer cameras. It can be seen from Figure 10b that the resolution of the object decreases as the distance increases, according to Formula (12):

(12) $$\frac{f}{D}=\frac{\mathrm{pixel}}{S},$$

where f is the focal length, D is the photographic distance, pixel is the size of each image element of the image, and S is the field distance represented by an image element.

As the distance increases, the actual distance of an object represented by a pixel becomes larger, which means that the accuracy decreases. The accuracy can reach 30 cm at 500 m, which meets the requirements for the calculation of slope parameters in open-pit mines and can be used for early warnings on slopes.

#### 3.1.2. Focal length, Field of View Angle and Quantity Analysis

Assuming that the distance was 200 m, we analyzed the quantity of cameras and object resolution results for different focal lengths (2.8–25 mm) and field of view angle conditions.

The calculation result is shown in Table 2. It can be seen that as the focal length increases, the field of view angle decreases accordingly and the quantity of cameras required gradually increases. This is because, as the field of view angle decreases, the sensing range of the camera head decreases, and more camera sensors are needed to meet the coverage requirements. In addition, it can be seen that the accuracy increases as the focal length increases, because, as the focal length increases, the range in which each pixel represents an object becomes smaller at the same distance, allowing the accuracy to increase. With a camera focal length of 2.8 mm, the accuracy can be achieved at a distance of 34 cm at 200 m.

Commonly, for open-pit mine slope damage alerts, the resolution of the monitoring should not be less than 50 cm. Most open-pit mines have high-definition cameras which can be fit for this requirement, but only be used for manual monitoring. In addition, the current deployment of their cameras is not suitable for slope monitoring. They need to deploy more cameras facing the mine slope if they want to realize slope damage risk monitoring. Through our OCD4M algorithm, we can make random deployments optimal. The algorithm can calculate the minimum quantity of cameras needed to achieve the large overlap and full coverage required to make the most of the mine’s video and multimedia resources.

### 3.2. Field Testing and Demonstration

**Figure 11:**
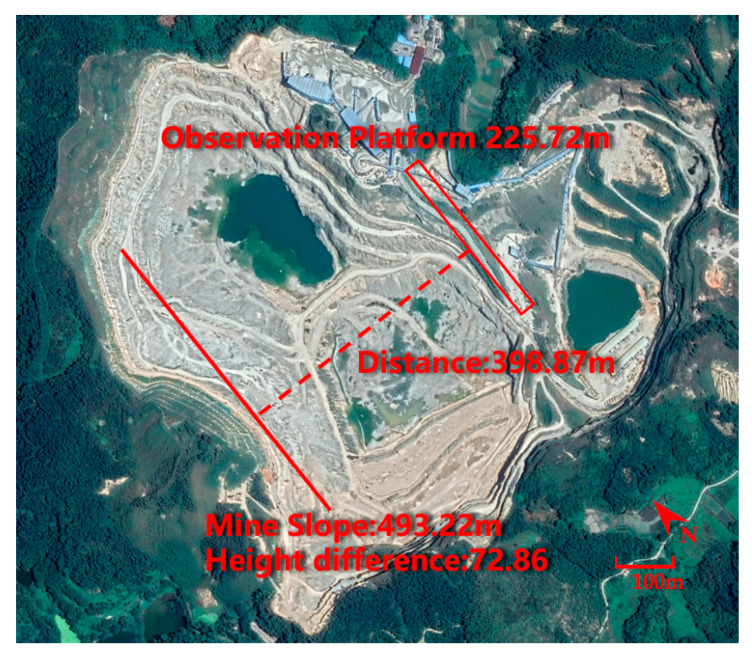
Study field located in Shunxing Quarry in Guangzhou, Guangdong, China.

The testing field is the Shunxing Quarry in Guangzhou, Guangdong, China, which is located at (113.518859 E, 23.405858 N), as shown in Figure 11. This is a medium and typical open-pit mine. The observed slope length of the mine is 493.22 m. The length of the mining platform is 225.72 m, and the slope height difference is 72.86 m. The distance (that is, the photography distance) is 398.97 m. The proposed camera model is HIKVISION DS-IPC-B12H-I with an 8 mm focal length, 1/2.7” sensor size, 32.69° field of view angle, and 24.81° vertical angle. Mine parameters, camera parameters, etc. were input into the OCD4M algorithm to calculate the minimum quantity of cameras and the coordinates of the monitoring points. A network of cameras was built in the field according to the coordinates and the 3D monitoring of the mine was automated without human participation by the data processing system we have developed. Given that we were working at the decimeter level of accuracy, we measured the slope of the open-pit, the height and width of the mining benches to be measured, and the volume of mining to be counted.

**Figure 12:**
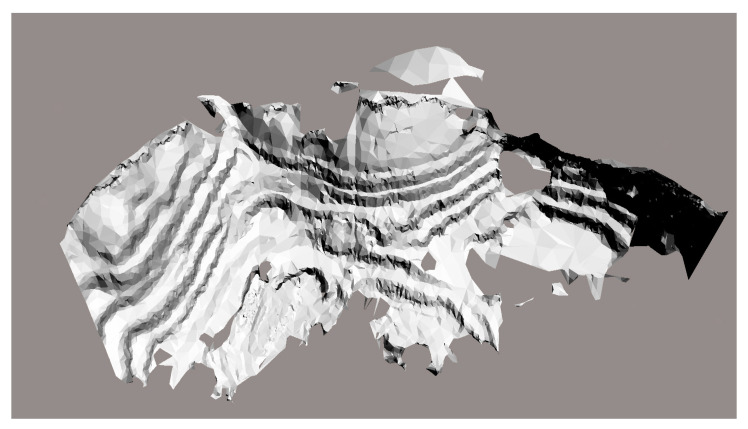
3D model of the study field.

**Figure 13:**
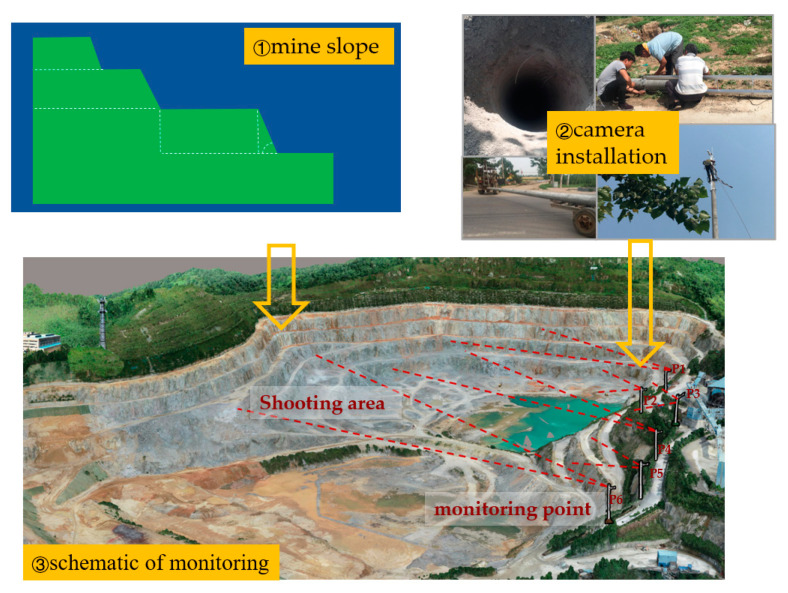
Schematic diagram of the camera field deployment. ① Schematic view of the slope of the mine. ② Pictures of the field installation, including pole tower transportation, drilling and camera installation, etc. ③ Schematic view of the Shunxing Quarry monitoring.

In the field, GPS sampling was used to locate camera points and set up the cameras. Multiple photos extracted from camera videos were prepared as the inputs. We used Smart3D software for the data preprocessing, aerial triangulation, dense matching of oblique images, DSM point cloud generation, triangulated irregular network (TIN) construction, texture mapping, model modification, and other processes, in order to produce a realistic 3D model, as Figure 12 and Figure 13 show.

**Figure 14:**
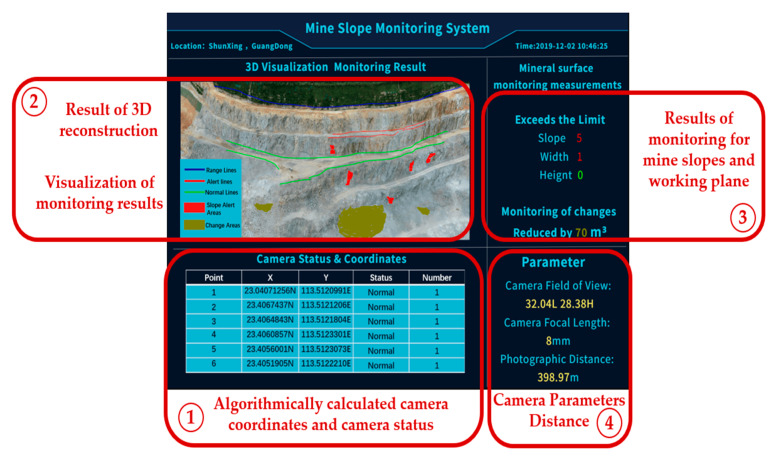
Software interfaces of the demonstration system.

Figure 14 illustrates the open-pit mine slope monitoring system which we developed for the visualization of the results of the algorithm and the early warnings during the safety monitoring. This can be used to generate the field camera deployment solution, analyze the slope of the mine, and measure the width and height of the working bench to assess and construct warnings about mine slope damage risks. It also integrates 3D reconstruction, 3D monitoring, and 3D visualization of the mine slope.

- The modular ① shows the number of camera points and their coordinates calculated by the algorithm and also indicates the camera status.
- The modular ② shows the result of the 3D reconstruction based on the camera photos and visualizes the mine plane and slope monitoring.
- The modular ③ shows statistical results of the monitoring of the mine plane risks which are related to slope damage indicators.
- The modular ④ shows the parameters of the camera and the monitoring distance.

As calculated by the OCD4M algorithm, the camera sensing range is between 234.62 and 290.58 m, the minimum quantity of cameras is six, and the coordinate results are shown in Table 3. In the given conditions, these six monitoring point coordinates are the best locations for deploying the cameras to monitor their opposite slopes.

**Figure 15:**
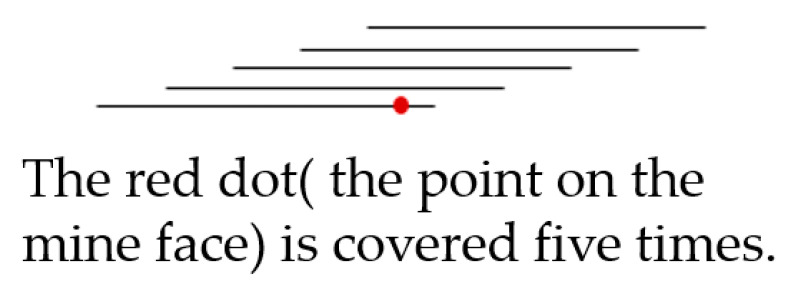
Relative overlap (80%), where each point is covered by five photos at least.

Zhang et al. [33] explored the relationship between overlap and accuracy. They assumed that each point is covered by photos five times, as shown in Figure 15. When the overlap is more than 80%, the accuracy is improved, but the speed of accuracy improvement is slowed down.

**Figure 16:**
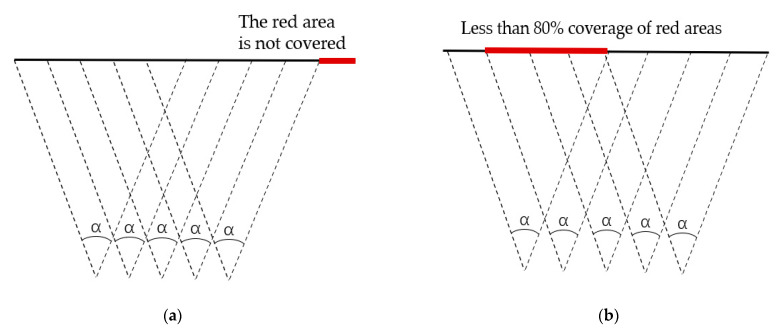
Comparison of different results when lacking one of the conditions: (**a**) 80% overlap at five cameras, no full coverage, and (**b**) full coverage at five cameras, overlap < 80%.

Considering the engineering practice, using more cameras is not conducive to cost control, and 80% overlap is a relatively reasonable choice in terms of the accuracy and engineering practice. Figure 16 shows a comparison of different results when lacking one of the conditions.

For verifying the result of the algorithm, we conducted a comparison test by reducing and increasing the quantity of cameras. The comparison of overlap and coverage length in conditions of different quantities is shown in Table 4. When the quantity of cameras is less than six, the camera monitoring cannot cover the whole mine surface with regard to overlap, and the overlap between adjacent photos will be less than 80%.

As shown in Table 4, meeting both requirements of the 493.2m photography distance and the 80% overlap, six cameras are needed to be deployed at least. Considering the actual engineering costs, using more cameras means higher cost. This proves that the deployment result (in Table 3) calculated by our OCD4M algorithm is relatively reasonable for this study case.

## 4. Discussion and Conclusions

In summary, the OCD4M algorithm is proposed for the deployment of camera sensor networks for slope monitoring to achieve the minimum quantity of cameras and obtain the deployment location coordinates, in order to optimize the deployment, enabling 3D monitoring capabilities and making full use of the multimedia data obtained from the cameras in the open-pit mine. We have conducted experimental validation with the simulated conditions of quantity, view angle, and focal length of cameras, at different monitoring distances. The OCD4M algorithm was tested in the medium-sized mine field, using Hikvision DS-IPC-B12H-I model 8 mm focal length cameras for mine surfaces photography and reconstructed in Smart3D software. The field test result shows that the accuracy of 30 cm can be achieved at the monitoring distance of 500 m. We also developed the visualization system software, through which the camera deployment scheme for the mine scenario can be generated automatically. According to the result of the algorithm, 3D monitoring of the working platform (e.g., calculating slope angle, height and width of the mine bench) can be realized at the decimeter level.

There are some considerations need to be emphasized in terms of deployment process, application scenario elaboration, engineering costs and limitations of the method. Since the physical deployment of control points is an unstable and costly solution for actual mining work, a high precision calibration of the camera is an important task [34]. Our method guarantees decimeter-level accuracy at 500 m monitoring distance without control points; thus, our method allows for decimeter resolution level safety monitoring in the open-pit mines, such as bench damage and interslope risk monitoring. However, overall landslide monitoring always needs a millimeter scale resolution, which no current camera model on the market can provide, especially when the monitoring slope is further than 500 m away. Due to topographical and other shading issues at the mine site, the use of front-to-surface photography may result in missing images in some areas that cannot be reconstructed in 3D, so the observation platform should be selected with due consideration of whether the area to be observed can be captured by all of the corresponding cameras. Considering the actual cost of the project, the low-cost solution of camera photogrammetry is easy to accept for small and medium-sized mines [7,21]. Additionally, our solution allows for automated monitoring after deployment, which also reduces the investment in manpower costs for the mine. It is more efficient than 3D laser scanning and traditional manual-based measurements. In the case of large mines, where the mines are large, distant, or complex, more cameras are required to ensure coverage and higher quality cameras to ensure accuracy, which can lead to increased costs, which are acceptable for the revenue of large mines.

In addition, the algorithm can be used for the calculation of other slope deployment scenarios (e.g., modeling of cultural heritage objects [35], 3D robot localization [36], monitoring coastal morphology [37], etc.). The algorithm can be improved by considering more photogrammetric geometry factors (e.g., the angle of intersection, the length of the photographic baseline, etc.) to optimize deployment scenarios for obtaining higher measurement accuracy, and by considering the mine topography and the actual deployable location of the mine to perform more complex deployment scenario calculations. The next step of the study will focus on the identification and warn of landslide areas using smart video image recognition based on the deployed system, so that the deployment algorithm can serve both monitoring of slope collapse risk and identifying landslide areas.

## Figures and Tables

**Table 1:** Characteristics and application scope of the main monitoring methods employed for open-pit mine slopes.

Monitoring Methods	Efficiency	Monitoring Cycle	Expenses (Estimate)	Accuracy
Traditional geodesic methods	Low efficiency (nonautomatic, restricted by terrain access and climate)	Depending on the monitoring task (ranging from one day to six months)	High costs (USD 2000 equipment costs + high labor costs)	High accuracy (millimeter, submillimeter)
GPS technology	High efficiency (automatic)	Real time	High costs (usually USD > 500,000 initial investment)	High accuracy (millimeter)
3D laser scanning technology	Medium efficiency (semiautomatic)	Depending on the monitoring task (ranging from one day to six months)	High costs (USD > 150,000 equipment costs + labor costs)	high accuracy (millimeter, centimeter)
Measuring robot technology	Medium efficiency (automatic, restricted by terrain access conditions)	Near real time	High costs, (USD > 150,000 equipment costs)	High accuracy (millimeter)
RS technology	Medium efficiency (semiautomatic)	Based on the revisit cycle of RS satellites (>4 days)	Low costs (USD > 3000 a view)	Low accuracy (decimeter, meter)
InSAR	Medium efficiency (semiautomatic)	Based on the revisit cycle of RS satellites (>11 days)	Low costs (USD > 7000 a view)	High accuracy (millimeter)
UAV photogrammetry	Medium efficiency (semiautomatic)	Depending on the monitoring task (ranging from one week to six months)	Low costs (USD > 4000 equipment costs + labor costs)	Medium accuracy (centimeter)
Digital close-up photogrammetry	High efficiency (automatic)	Near real time	Low costs (USD 1500 a monitoring camera point)	Medium accuracy (centimeter, decimeter)

**Table 2:** Quantity of cameras and accuracy calculated by different focal lengths and view angles.

Focal Length (mm)	Viewing Angle (°)	Photographic Range (m)	Quantity of Cameras	Accuracy (cm)
2.8	79.93	335.22	3	34
4	60.79	234.63	6	24
6	42.72	156.44	11	16
8	32.69	117.31	17	12
12	22.12	78.19	27	8
16	16.68	58.64	38	6
25	10.72	37.50	62	4

**Table 3:** Camera coordinates for the 6 different monitoring points shown in Figure 13.

Point	X	Y
1	23.0407125 N	113.5120991 E
2	23.4067437 N	113.5121206 E
3	23.4064843 N	113.5121804 E
4	23.4060857 N	113.5123301 E
5	23.4056001 N	113.5123073 E
6	23.4051905 N	113.5122210 E

**Table 4:** Overlap and coverage length for different quantity of cameras.

Quantity of Cameras	Overlap at Full Coverage (%)	Coverage at 80% Overlap(m)
4	72.44	422.31
5	77.96	469.24
6	81.63	516.16
7	84.25	563.08
8	86.22	610.01

## Data Availability

Data sharing not applicable.
